# Expression patterns of core metabolic genes and elevated intracellular ROS confer drug tolerance in *Staphylococcus aureus*

**DOI:** 10.1128/spectrum.01868-25

**Published:** 2026-02-10

**Authors:** Jiahao Liu, Yeming Li, Haodong Liu, Shunhua Yan, Haoyi Jia, Yuxuan Yang, Wenting Hu, Jingjia Wang, Wenfeng Li, Huping Xue, Xin Zhao, Long Li, Pilong Liu

**Affiliations:** 1 College of Animal Science and Technology, Northwest A&F University 546344, Yangling, Shaanxi, People’s Republic of China; 2 College of Animal Engineering, Key Laboratory for Efficient Ruminant Breeding Technology of Higher Education Institutions in Shaanxi Province, Yangling Vocational and Technical College 438730, Yangling, Shaanxi, China; 3 Department of Animal Science, McGill University 317104, Sainte-Anne-de-Bellevue, Québec, Canada; Yan'an University, Yan'an, Shaanxi, China

**Keywords:** antibiotic tolerance, expression pattern, *Staphylococcus aureus*, metabolic regulation, ROS

## Abstract

*Staphylococcus aureus* exhibits remarkable tolerance to antibiotic stress, facilitated by a complex network of cellular responses and metabolism controlled by numerous gene expression patterns that can be rapidly remodeled. This tolerance can lead to treatment failure and the emergence of antibiotic resistance. However, the expression patterns of these genes caused by metabolic alterations driving antibiotic tolerance remain poorly understood. Our objective was to identify the core metabolic genes involved in the development of tolerance. Using proteomic analysis and gene complementation assays, we found that seven tolerant isolates shared similar protein expression profiles and mechanisms for tolerance. Seven metabolic genes, including *NWMN_0676-0677*, *opuCB*, *gltD*, *adhE*, *clpP*, and *rarA*, were confirmed as major contributors to tolerance. Notably, these genes were linked to elevated intracellular reactive oxygen species (ROS) levels in drug-tolerant strains. Treatment with ROS scavengers increased the sensitivity of these strains to antibiotics. These results demonstrate that changes in the expression of metabolic genes play a crucial role in the development of drug tolerance, and the regulation of ROS metabolism may be central to the broader metabolic alterations in drug-tolerant bacteria.

**IMPORTANCE:**

*S. aureus* poses a major public health threat due to its remarkable ability to develop antibiotic tolerance, often leading to treatment failure and resistance emergence. This study provides critical insights into the underlying metabolic mechanisms. Proteomic analysis revealed that different genetic mutations in tolerant isolates converged on similar gene expression changes, which directly impacted the tolerance phenotype. Notably, the tolerant strains exhibited elevated intracellular reactive oxygen species (ROS) levels, and ROS scavenger treatment increased their antibiotic susceptibility. These findings demonstrate that shifts in core metabolic gene expression are pivotal for *S. aureus* to withstand antibiotic stress, with ROS metabolism regulation being a central component of the broader metabolic adaptations conferring drug tolerance. Understanding these metabolic underpinnings is crucial for developing more effective treatments against persistent, tolerant *S. aureus* infections. The identified metabolic targets and ROS-modulating approaches offer promising strategies to combat escalating antibiotic resistance.

## INTRODUCTION

As one of the primary contributors to significant morbidity and mortality, antibiotic tolerance has attracted considerable attention in recent years (1). However, the inconsistency of genetic mutations with tolerance phenotype to a specific antibiotic suggests that there may be mechanisms beyond mutations that remain to be explored. Unlike antibiotic resistance, tolerance does not involve mutations of the antibiotic-targeting genes; it enhances the survival of bacterial populations under environmental stress (2). Once the antibiotic concentration decreases or is eliminated, surviving cells can repopulate, leading to relapsing chronic infections (3). More importantly, the emergence of antibiotic tolerance enables bacteria to evolve antibiotic-resistant phenotypes (4). Thus, having a thorough understanding of the mechanisms underlying tolerance formation is crucial for the development of new antibacterial therapies.

*Staphylococcus aureus* is a common pathogen associated with serious diseases, including bacteremia, endocarditis, and various related infections (5). Treatment failure due to *S. aureus* occurs in approximately one in four patients, often resulting in frequent recurrences and high mortality rates (6). Resensitizing tolerant *S. aureus* to existing antibiotics has become a key research focus. Current studies on *S. aureus* tolerance primarily concentrate on identifying genetic factors associated with phenotypic tolerance formation and the environmental triggers involved (7). However, the inconsistency of genetic mutations with tolerance phenotype to a specific antibiotic suggests that there may be other mechanisms beyond mutations.

The important role of metabolic remodeling in the development of bacterial tolerance has become increasingly recognized. Metabolic changes in bacterial cells are a key physiological factor that influences intrinsic susceptibility to antibiotics (8). Indeed, the metabolic state is increasingly recognized as a critical determinant of antibiotic tolerance, where bacteria survive transiently high antibiotic concentrations without genetic resistance mechanisms. For instance, phenomena such as bacterial persistence are often linked to dormant metabolic states and ATP depletion (9, 10). Specific metabolic pathways, such as the TCA cycle, have been shown to have heterogeneous expression, contributing to the formation of persister cells (10). While some metabolites, such as α-Ketoglutarate (11) and exogenous glutamate (12), can potentiate antibiotic killing, the underlying metabolic reprogramming that allows bacteria to tolerate antibiotics often involves a broader reduction in metabolic activity or shifts towards specific survival-oriented metabolic states (13).

Despite the diversity of mutations associated with tolerance, we hypothesized that these different evolutionary trajectories would ultimately converge on common, non-heritable changes at the level of gene expression. We further hypothesized that this convergent expression reprogramming establishes a shared physiological state—specifically, an altered metabolic and redox environment—that is central to the tolerant phenotype. This study, therefore, aims to identify the core metabolic gene set in independently evolved *S. aureus* tolerant lineages and elucidate how their expression patterns are reprogrammed to regulate cellular redox balance and ultimately confer multidrug tolerance.

## RESULTS

### Convergent metabolic adaptations underpin antibiotic tolerance

**Fig 1:**
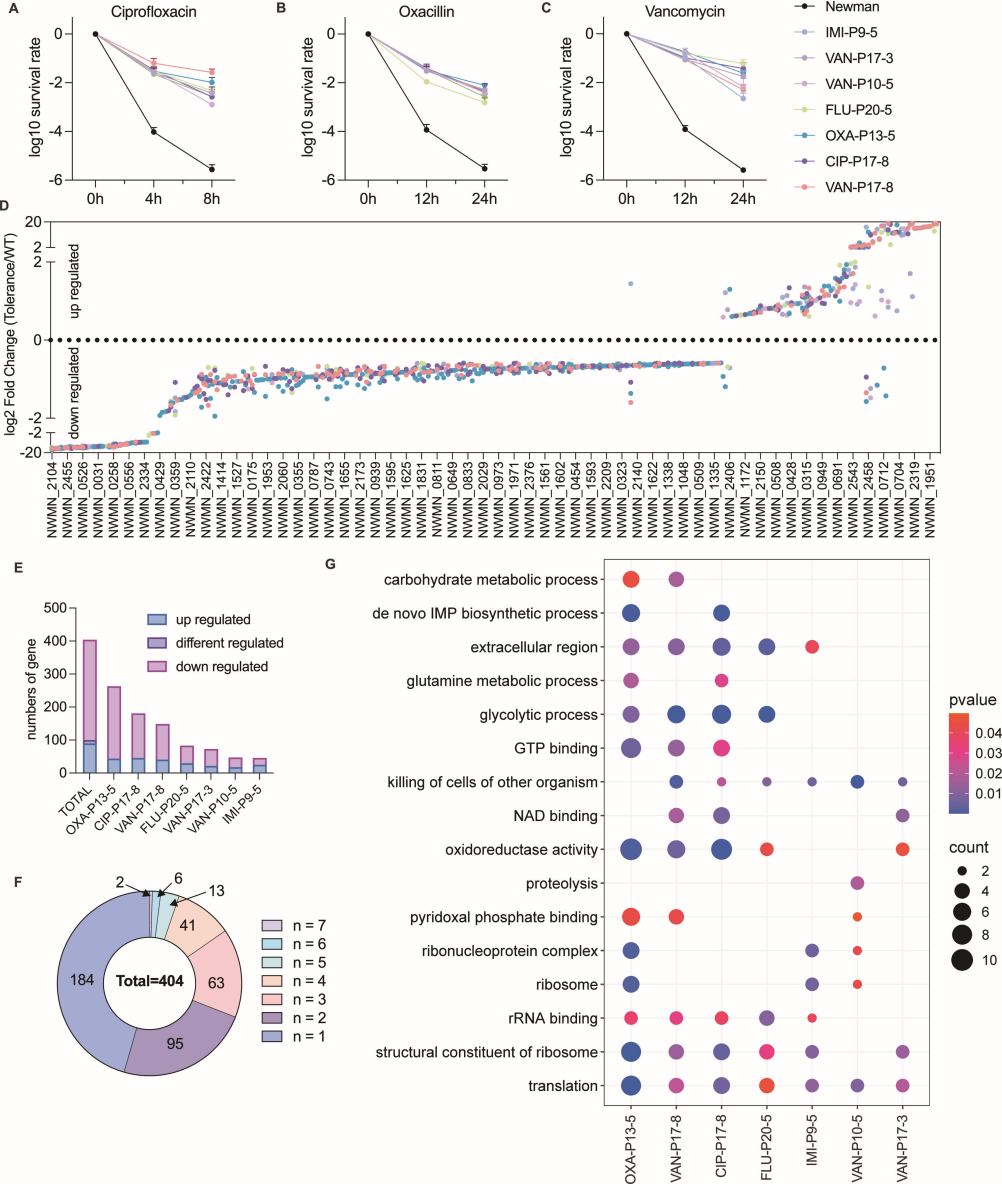
Differentially expressed proteins in tolerant *S. aureus* strains compared to wild type. (**A–C**) Seven tolerant strains were killed by diverse antimicrobials. Survival of exponentially growing wild-type and all mutant cells was measured after treatment with ciprofloxacin (20× MIC for 8 h), oxacillin, and vancomycin (20× MIC for 24 h). Data are averages of three biological replicates; error bars indicate SEM. (**D**) A total of 404 DEPs were identified through proteomic analysis of tolerant strains compared to wild type. Each circle on the plot represents the average log_2_ fold change from three biological replicates, with DEPs ordered by ascending log_2_ fold change. Different colored circles correspond to the seven tolerant strains. (**E**) Bar graphs display the number of DEPs that showed upregulation (blue), downregulation (pink), or differing expression trends (purple) across the seven proteomes. (**F**) The pie chart illustrates the frequency of DEPs across the seven proteomes (e.g., there were 63 DEPs captured in any three proteomes). (**G**) Gene ontology (GO) enrichment analysis of all DEPs identified in the seven tolerant strains was conducted using the clusterProfiler R package. Only GO terms with a *P*-value below 0.05 were considered significantly enriched.

In our previous study, *S. aureus* strain Newman was evolved in different antibiotics, and the evolved strains were isolated (14). Then, the multi-tolerance phenotype of these strains was assessed using three different types of antibiotics (ciprofloxacin, oxacillin, and vancomycin), chosen for their distinct bactericidal mechanisms (Fig. 1A through C). There were different genetic mutations in the genomes of these strains (Table S1). However, unlike previous work (15), slow growth or longer lag time did not account for tolerance, as growth rates were similar for the majority of tolerant strains and wild-type cells (Fig. S1). Based on these findings, our subsequent work focused on investigating all seven tolerant strains to uncover the mechanisms underlying their tolerance.

To identify the stable, inherent physiological changes that define the multi-drug tolerant phenotype, we performed quantitative proteomic analysis comparing the seven evolved tolerant strains to the ancestral Newman wild-type strain. All strains were cultured under identical, antibiotic-free conditions in their stationary growth phase. This experimental design was deliberately chosen to isolate the basal proteomic alterations that have become fixed during the evolution of tolerance. This experimental design was chosen specifically to isolate the basal proteomic alterations that have accumulated during the evolution of tolerance, rather than to capture the acute transcriptional response to antibiotic exposure. Then 404 differentially expressed proteins (DEPs) were identified between these seven strains and wild type (Fig. 1D). Ninety of these were consistently upregulated, and 304 were downregulated across all seven strains (Fig. 1E). Interestingly, 125 DEPs exhibited the same expression trend (either upregulated or downregulated) in at least three of the cross-tolerant strains, while 62 DEPs shared this trend in at least four strains, 21 DEPs shared in at least five strains, 8 DEPs shared in at least six strains, and 2 DEPs shared in all seven strains (Fig. 1F). These findings suggest that there may be similar or identical expression changes in metabolic genes responsible for bacterial adaptation to different antibiotics.

We next analyzed the DEPs using gene ontology (GO) enrichment analysis. Hypergeometric test of all DEPs revealed significant enrichment in molecular functions such as oxidoreductase activity, NAD binding, and GTP binding, all of which play crucial roles in cellular metabolism (Fig. 1G). Importantly, various metabolic processes, including glycolysis, carbohydrate metabolism, and *de novo* IMP biosynthesis, were enriched across some of the strains, suggesting that bacteria may alter metabolic fluxes to maintain a tolerance phenotype (16, 17). Furthermore, the DEPs were associated with transcription- and translation-related processes, including translation, ribonucleoprotein complex formation, ribosomal structure, rRNA binding, and extracellular region (Fig. 1G). Since transcription and translation are among the most energy-intensive processes in the cell (18, 19), their alteration might cause shifts in metabolic fluxes, which are key to cross-tolerance (18). Overall, our results indicate that tolerant strains evolved from different drugs share similar protein expression patterns, likely driven by changes in metabolism.

### Core metabolic genes identified as critical mediators of multidrug tolerance

**Fig 2:**
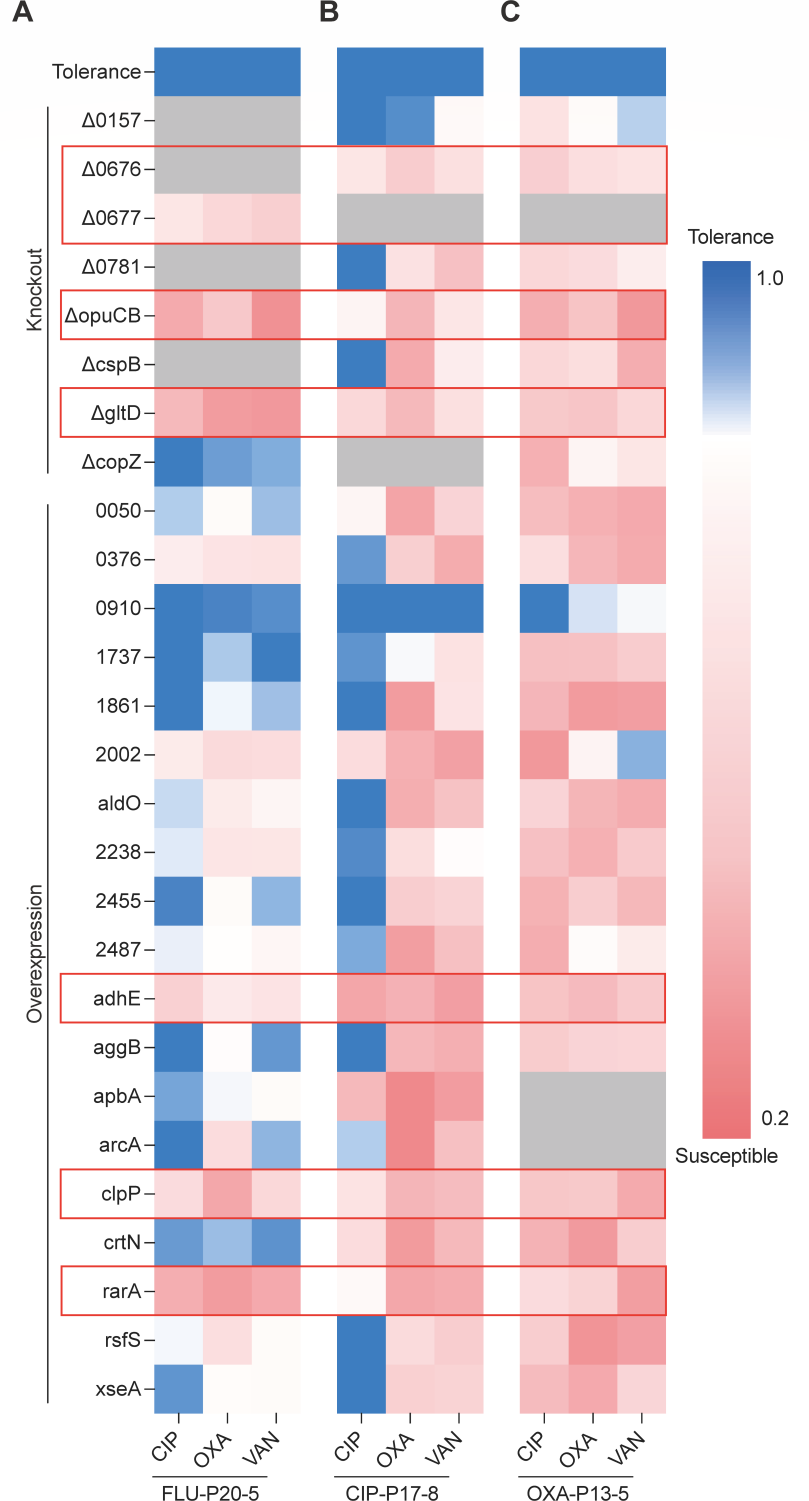
The roles of metabolic gene expressions in the tolerance formation. (**A–C**) Heatmap depicting antibiotic tolerance profiles of tolerant *S. aureus* strains and derived mutants. This heatmap illustrates the relative TI rate of the antibiotic-tolerant *S. aureus* strains FLU-P20-5, CIP-P17-8, and OXA-P13-5, along with their corresponding gene deletion or overexpression mutants, when exposed to different antibiotics. The relative TI rate is calculated by dividing the tolerance index (TI) of each mutant strain by the TI of its parental tolerant strain. The color gradients represent the relative TI rates, with white and pink indicating lower tolerance or non-tolerance, blue denoting higher tolerance, and gray cells representing cases where mutants could not be successfully generated in the corresponding strain background. The red frames indicate genes that are crucial to the development of the multidrug tolerance phenotype. This visualization allows for a comprehensive comparison of the tolerance phenotypes across the different tolerant strains and their engineered mutants, revealing the strain-specific and antibiotic-dependent impacts of modulating the expression of key metabolic genes.

To confirm the effect of metabolic alteration on multidrug tolerance, 27 genes were selected for further investigation, based on factors such as statistical significance, fold changes in expression, and the frequency of DEPs observed across tolerant strains (Table 1). We created deletion (if the gene is upregulated) or overexpression (if the gene is downregulated) mutants of these genes in three tolerant strains (FLU-P20-5, CIP-P17-8, and OXA-P13-5). The efficiency of gene deletion or overexpression was validated using quantitative real-time PCR (Fig. S2). The tolerance of these evolved strains and mutants to antibiotics was tested and represented by the tolerance index (TI) (Fig. S3). The relative TI rate of each strain to each antibiotic was visualized by heatmap (Fig. 2A through C). Most mutants with genetic manipulation showed increased susceptibility to one of the three antibiotics. These results confirm that changes in the expression of metabolism-related genes have a direct impact on the multiple-drug-tolerant phenotype.

**TABLE 1:** Different expressed proteins displayed similar expression patterns in at least three tolerant strains

Protein names	Description	Log_2_ fold change						
		VAN-P17-3	VAN-P10-5	IMI-P9-5	FLU-P20-5	OXA-P13-5	CIP-P17-8	VAN-P17-8
RarA; NWMN_1529	DNA replication-associated recombination protein	–^*a*^	–	–	–	−15.44336587	−15.10630768	−14.92174731
XseA; NWMN_1428	Exodeoxyribonuclease 7 large subunit	−13.70084814	–	–	–	−14.85324885	−13.85043543	−13.91849427
ClpP; NWMN_0736	Clp protease proteolytic subunit	−1.386862342	−1.05219633	−0.971296466	−1.117196139	−1.06609624	−1.370162308	−1.110429498
RsfS; NWMN_1494	Ribosomal silencing factor	−14.32077225	−13.56965074	−14.73122003	−13.69908001	–	–	–
NWMN_0050	Fatty acid metabolic	–	–	–	−14.45409526	−15.31170537	−16.16245469	–
ApbA; NWMN_2342	Pantothenate biosynthesis	–	–	–	–	−11.35750103	−10.44356235	−10.36912808
ArcA; NWMN_2534	Arginine metabolism	–	–	–	–	−12.48333182	−13.21345873	−12.84436274
CrtN; NWMN_2461	Carotenoid biosynthesis	–	–	–	−15.10800757	−15.4793984	−15.01574703	−15.41316682
AdhE; NWMN_0094	Aldehyde-alcohol dehydrogenase	–	–	–	−16.09168747	–	−16.30338655	−15.90029698
AldO; NWMN_2104	Aldo/keto reductase	−18.06140546	−17.66737362	–	−15.32453667	−17.39760846	−16.20811906	−16.45864965
GltD; NWMN_0437	Glutamate synthase	−1.241125762	−1.480821184	–	–	11.42955781	10.68070494	–
NWMN_0781	ABC transporter permease	3.098540293	2.001283306	2.776921384	–	2.044148843	–	4.05956527
CopZ; NWMN_2458	Copper chaperone	2.242423749	–	–	1.36130114	12.83810045	2.865556108	3.720648768
OpuCB; NWMN_2346	Glycine betaine/carnitine ABC transporter permease	–	–	–	−0.72572966	−1.08736179	−0.715563079	−0.867895839
NWMN_0157	Putative transferase protein	–	–	–	16.52974879	15.03926317	14.3127611	16.8597739
NWMN_0376	Unknown	–	−15.66926344	−15.66946103	−15.29175023	−15.99078908	–	–
NWMN_0676	Two-component system	–	–	–	20.12056796	20.37201536	18.04187299	19.98707966
NWMN_0677	Two-component system	–	–	–	7.668312875	8.10983997	7.880377217	7.968575297
NWMN_0910	Putative acetyltransferase	−11.84198006	−11.55155868	–	–	−12.00427606	−12.34680065	−12.25203551
NWMN_1737	Unknown	−0.809765548	−0.707198994	−0.903465452	−0.90526548	−0.864465483	−1.047465326	−0.669665695
NWMN_1861	Putative phage protein	−0.767565169	–	−0.856831681	−1.343164461	−0.757365097	−1.244397809	−0.850998295
NWMN_2002	Unknown	−14.97404817	–	–	–	−15.43599976	−14.41157327	–
NWMN_2238	MOSC domain protein	–	−14.36454015	–	–	−12.72046375	−12.15118823	−14.10835875
NWMN_2455	Putative acetyltransferase	−15.48116675	–	–	−15.28167237	−15.0639763	−15.36333458	−15.53544027
NWMN_2487	Putative fructosamine kinase	–	–	–	−1.556379649	−1.6055791	−1.714245152	−1.665812198
AggB; NWMN_0526	Glycosyl transferase	−13.73984375	–	−14.89748082	−14.9105477	−15.72682838	−15.49349824	−15.10319912
CspB; NWMN_2605	Cold shock protein	2.050932553	0.990059492	–	–	5.220877411	2.374666922	–

^*a*^ –, not detected.

As expected, the antibacterial activity of different antibiotics against mutant strains varied. Particularly, ciprofloxacin showed stronger bactericidal activity against mutants derived from OXA-P13-5 than those from FLU-P20-5 and CIP-P17-8 (Fig. 2A through C). Similarly, the effects of these DEPs on the tolerant phenotype exhibited a certain degree of strain specificity. Fortunately, some DEPs have phenotypic effects that are independent of both the strain and the antibiotic. We believe that these DEPs play a central role in the development of multi-drug tolerance in bacteria. These core genes, involved in pathways such as two-component sensing (*NWMN_0676/7*), osmolyte transport (*opuCB*), glutamate synthesis (*gltD*), redox balance (*adhE*), proteolysis (*clpP*), and DNA repair/recombination (*rarA*). Notably, NWMN_0676 and NWMN_0677, also known as SaeQ and SaeP, function as auxiliary components of the two-component system SaeRS. The deletion of either SaeP or SaeQ disrupts the dephosphorylation of P-SaeR, thereby altering the signal transduction mediated by SaeRS (20). Therefore, we still suggest that the loss of either gene results in the absence of the multidrug tolerance phenotype, although specific mutants could not be generated.

The identification of these core genes, including the redox-active enzyme AdhE (alcohol dehydrogenase), provided functional validation linking specific metabolic pathways to tolerance. This finding, combined with our initial proteomic analysis, which revealed altered expression of numerous proteins with oxidoreductase activity across the tolerant strains (Fig. 1D and G), strongly pointed towards cellular redox imbalance as a potentially critical and convergent feature underlying the observed multidrug tolerance.

### Redox homeostasis underlies broad-spectrum antibiotic tolerance

**Fig 3:**
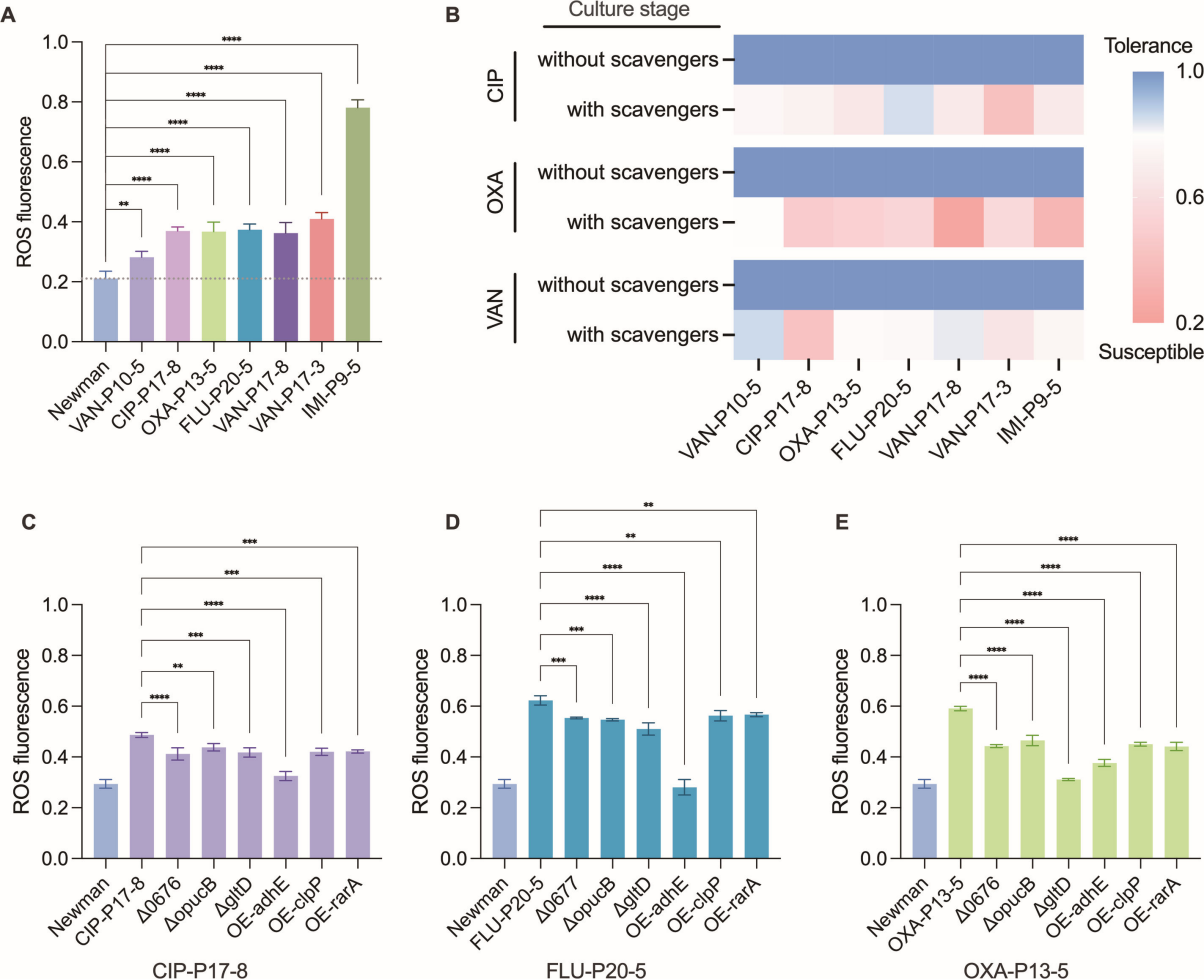
The relationship between intracellular ROS and tolerance formation. (**A**) ROS fluorescence in seven *S*. *aureus* Newman-derived tolerant strains compared to Newman. Bacteria strains were grown overnight, diluted OD_600_ to 0.5 by using PBS, and treated with DCFH-DA (10 μM) for 30 min. Then, intensity quantification by a fluorescence microplate reader for staining in cultures. (**B**) Percent survival rate of *S. aureus* Newman and all tolerant strains in the presence of various antibiotics with or without ROS scavengers (100 mM thiourea and 1 mM bipyridine). The survival rate is measured in different time (ciprofloxacin 8 h; oxacillin and vancomycin 24 h). (**C–E**) ROS level in six core gene mutants compared to their homologous tolerant strains. Data are means ± SD from three biological replicates. **P ≤ 0.01; ***P ≤ 0.001; ****P ≤ 0.0001.

Indeed, the proteomic data highlighted 10 DEPs encoding oxidoreductase activities across the drug-tolerant strains (Fig. 1D), suggesting that the formation of tolerance may be associated with alterations in bacterial redox states. ROS are key contributors to altered cellular redox potential (21). Several previous studies have found that bacteria generate a significant amount of ROS during the process of antibiotic killing (22–24). Then, we measured ROS levels in stationary-phase cultures using the fluorescent dye DCFH-DA [5(6)-carboxy-2′,7′-dichlorodihydrofluorescein diacetate]. Interestingly, our results indicated elevated ROS levels in all tolerant strains (Fig. 3A). To determine if this elevated basal ROS level was functionally important for the tolerant phenotype during antibiotic challenge, we treated the strains with ROS scavengers. As shown in Fig. 3B, the addition of scavengers dramatically reduced the survival of all tolerant strains over the entire course of antibiotic treatment (8 h for ciprofloxacin; 24 h for oxacillin and vancomycin), effectively re-sensitizing them to the antibiotics. This result confirms that the constitutively higher intracellular ROS level is not merely a correlative biomarker but a key functional contributor to the maintenance of drug tolerance under prolonged antibiotic stress. To determine if these seven core genes directly influence intracellular ROS, we measured ROS levels in the respective gene deletion or overexpression mutants. Unexpectedly, the deletion of *NWMN_0676/7*, *opuCB,* or *gltD* and the overexpression of *adhE*, *clpP,* or *rarA* all led to a decrease in ROS levels (Fig. 3C). This result implies that bacteria may modulate intracellular ROS levels by regulating the expression of metabolic genes and evolve adaptations to antibiotics.

## DISCUSSION

### Convergent proteomic reprogramming as a hallmark of evolved tolerance

This study illuminates a convergent metabolic and redox adaptation strategy in *S. aureus* that confers multidrug tolerance. A key finding is that despite diverse initial antibiotic selective pressures and distinct underlying genetic mutations in the evolved strains (Table S1), these lineages consistently converged upon similar alterations in their proteomic profiles. This observation challenges the paradigm that specific genetic mutations are the sole drivers of tolerant phenotypes. While numerous studies have identified genetic variations associated with antibiotic tolerance (23, 25, 26), our findings align with the growing understanding that such mutations are not always strictly necessary for the emergence of tolerance (27–29). Our experimental design, which analyzed the basal proteome of evolved strains in the absence of antibiotics, was deliberately chosen to isolate the stable, fixed physiological changes that define the tolerant state. This contrasts with proteomic studies that analyze the acute response to antibiotic stress (30), typically identifying the immediate upregulation of cell wall synthesis pathways and other direct stress responses. Our approach filtered out these transient responses, revealing a convergent reprogramming of gene expression patterns. Indeed, non-heritable tolerant states, such as persistence and phenotypic heterogeneity, underscore the importance of non-genetic mechanisms (2).

Our results strongly suggest that the development of drug tolerance in *S. aureus* is primarily orchestrated by dynamic reprogramming of gene expression patterns rather than by a fixed set of resistance-conferring mutations. Under antibiotic stress, phenotypes conferring a survival advantage, driven by these expression changes, can accumulate within the population, potentially reinforced by positive feedback mechanisms (29, 31, 32). When the environmental stressor is an antibiotic, the outcome of this adaptive feedback is the emergence of drug tolerance (33). The remarkable similarity in expression patterns across independently evolved tolerant strains, despite their different mutational landscapes, implies that *S. aureus* navigates the evolutionary path to tolerance through common alterations in fundamental cellular processes. This insight into the molecular underpinnings of antibiotic tolerance and its inherent plasticity carries significant therapeutic implications. Specifically, it suggests that targeting the regulatory networks governing metabolic gene expression, or the metabolic vulnerabilities arising from such expression changes, may represent a more effective and broadly applicable strategy for combating the emergence and persistence of drug-tolerant bacterial populations than approaches solely reliant on identifying and targeting specific genetic mutations associated with classical resistance (34).

### The pivotal role of core metabolic gene expression in driving multidrug tolerance

Building on the observation of convergent proteomic profiles, our investigation identified a core set of seven genes whose expression patterns were consistently altered across the tolerant isolates and were functionally validated as major contributors to the multidrug-tolerant phenotype. These genes include NWMN_0676 and NWMN_0677 (auxiliary components of the SaeRS two-component regulatory system), OpuCB (a permease for glycine betaine/carnitine transport, involved in osmotic stress response), GltD (a subunit of glutamate synthase, crucial for nitrogen metabolism and amino acid biosynthesis), AdhE (an alcohol-aldehyde dehydrogenase involved in fermentation and redox balancing), ClpP (the proteolytic subunit of the ATP-dependent Clp protease, central to protein quality control), and RarA (a DNA replication and repair protein). Our proteomic approach revealed that the evolution of tolerance is strongly correlated with convergent expression changes in these core genes, many of which are novel effectors tied to basic metabolism (e.g., GltD, AdhE, and ClpP) and regulated by the SaeRS system (NWMN_0676/7). We note that this mechanism is distinct from those observed in other adaptive evolution studies that utilized different selective pressures, such as the membrane-targeting antibiotic daptomycin or human serum (35, 36). Those studies demonstrated that bacteria preferentially evolved resistance via genetic mutations in specific regulatory components linked to the cell wall, particularly the WalKR and GraSR two-component systems. These findings underscore the complexity of tolerance acquisition, highlighting that both stable genetic variation and convergent gene expression reprogramming are crucial evolutionary strategies employed by *S. aureus* to survive antibiotic stress.

The targeted manipulation (deletion or overexpression) of these core genes in tolerant backgrounds directly impacted their susceptibility to multiple antibiotics, confirming that alterations in their expression are not mere correlates but functional determinants of the tolerant state. This direct, functionally validated correlation between protein expression and the tolerance phenotype in our evolved isolates presents a clear case of proteome-fitness linkage. We acknowledge that this finding is distinct from other important studies that have observed expression-fitness decoupling, a principle where changes in molecular abundance do not always correspond to a gene’s contribution to fitness (37). For instance, a relevant study in *S. aureus* biofilms by Freiberg et al. clearly demonstrated this decoupling: while arginine deiminase pathway proteins (e.g., ArcA) were upregulated under antibiotic stress, transposon sequencing (Tn-Seq) showed their genes had no significant fitness contribution (38). While such decoupling highlights the complexity of interpreting “omics” data, our own results provide a robust demonstration that for this core set of seven genes, the convergent proteomic alterations we identified are the functional drivers of the multidrug-tolerant phenotype. These findings underscore that bacterial adaptation to antibiotic stress involves a coordinated remodeling of diverse cellular pathways, encompassing signal transduction (20, 31), nutrient uptake (32, 33), central metabolism (12, 34, 39, 40), redox homeostasis (41, 42), protein turnover (43, 44), and DNA maintenance (45, 46). The consistent involvement of this core gene set across strains tolerant to different classes of antibiotics points toward a shared, fundamental adaptive strategy employed by *S. aureus* to withstand antibiotic killing.

### Elevated intracellular ROS: a pro-tolerance signal co-opted by evolved strains

A striking physiological feature shared by the drug-tolerant *S. aureus* strains was a significant elevation in intracellular ROS levels (Fig. 3A). Crucially, the enhanced survival of these tolerant strains under antibiotic challenge was substantially diminished by treatment with ROS scavengers, indicating a functional, pro-tolerance role for these elevated ROS levels (Fig. 3B). Further interrogation revealed that the expression changes in the identified core metabolic genes were directly linked to this altered redox state (Fig. 3C). This suggests that *S. aureus* may actively modulate its intracellular ROS environment by regulating the expression of these core genes as an adaptive strategy to antibiotic exposure.

The role of ROS in the context of antibiotic action is complex and often appears paradoxical. While numerous studies demonstrate that exposure to bactericidal antibiotics can trigger a surge in intracellular ROS, contributing to cellular damage and death (23, 24, 47), our findings, along with others (48, 49), suggest that ROS can also play a protective role. It is plausible that the sustained, moderately elevated ROS levels observed in our tolerant strains, resulting from specific metabolic reprogramming, function as critical signaling molecules. Such ROS signals could activate protective stress response pathways or modulate the expression of other metabolic genes, thereby promoting adaptive physiological states conducive to survival under antibiotic pressure (50, 51). This scenario contrasts sharply with conditions where acutely high, uncontrolled ROS bursts exceed cellular detoxification and repair capacities, leading to widespread macromolecular damage and cell death (22, 52, 53). This proposed context-dependent duality of ROS function represents a key hypothesis explaining its integral role in the nuanced bacterial stress response observed.

### Metabolic reprogramming: interplay with cellular bioenergetics and stress adaptation

Our findings resonate strongly with a growing body of literature implicating profound metabolic shifts, particularly concerning energy metabolism, in bacterial persistence and antibiotic tolerance. Seminal studies have demonstrated that reduced ATP levels, often driven by stochastic expression of enzymes in central metabolic pathways like the tricarboxylic acid (TCA) cycle, are a hallmark of persister cells and contribute significantly to their antibiotic recalcitrance (9, 10). Furthermore, ATP depletion has been shown to reduce translation efficiency, a critical factor in bacterial tolerance (54), an observation that aligns with the alterations in translation-related processes identified in our drug-tolerant *S. aureus* strains through proteomic analysis.

While our study did not directly quantify cellular energy charge, several lines of evidence point toward a significant alteration in bioenergetics within the tolerant strains. Proteomic analysis revealed the downregulation of proteins involved in pathways associated with ATP synthesis (Fig. S4). GO enrichment analysis also highlighted alterations in glycolysis and carbohydrate metabolism—processes intrinsically linked to cellular energy levels. The observed downregulation of energy-intensive processes, such as translation, further supports a potential adaptation toward a lower energy state or a re-prioritization of metabolic resource.

The interplay between ROS and cellular energy state is intricate; elevated ROS can impair ATP synthesis by damaging respiratory chain components, while a compromised energy metabolism, leading to electron transport chain stagnation, might itself foster increased ROS production. The elevated ROS levels in our tolerant strains could be both a consequence and a contributing factor to a potentially altered cellular energy state, creating a reinforcing feedback loop that promotes tolerance. Collectively, our findings strongly suggest that the metabolic reprogramming fundamental to the tolerant phenotype in *S. aureus* likely involves significant modulations in cellular bioenergetics, contributing to enhanced survival under antibiotic stress.

### Functional insights from specific core genes: ClpP and arginine metabolism

Among the core genes whose altered expression was linked to tolerance, *clpP* and *arcA* provide compelling examples of specific metabolic nodes influencing this phenotype. The gene *clpP*, encoding the proteolytic subunit of the Clp protease, was consistently downregulated across all seven tolerant strains. Our functional assays confirmed the importance of this downregulation, as overexpressing *clpP* in tolerant backgrounds rendered the cells more susceptible to antibiotics. This suggests that reduced basal ClpP-mediated proteolysis contributes to the maintenance of the multidrug-tolerant phenotype, perhaps by stabilizing proteins crucial for stress adaptation (55–59). This finding is intriguingly complementary to the work by Conlon et al., who demonstrated that pharmacological activation and dysregulation of ClpP by ADEP4 is a highly effective strategy for eradicating dormant *S. aureus* persister cells (60). In that context, ADEP4 converts ClpP into a non-specific, uncontrolled protease, forcing lethal self-digestion, a mechanism that bypasses the inactivity of conventional antibiotic targets in dormant cells. Thus, while a reduction in regulated ClpP activity may be part of a natural tolerance strategy, the ClpP protease itself represents a critical vulnerability that can be exploited to eliminate tolerant populations. The downregulation we observed might even render these tolerant cells particularly reliant on their remaining basal ClpP function or more susceptible to the dysregulating effects of ClpP activators.

Furthermore, our proteomic analysis identified altered expression of an arginine metabolism gene, *arcA* (*NWMN_2534*, encoding arginine deiminase), which was downregulated in three of our tolerant strains. Our result, informed by recent literature, confirms that the entire arginine metabolic network is a central hub for antibiotic tolerance. Indeed, the synthesis branch of this network, governed by genes such as *argF* and *argH*, represents a critical switch for inducing tolerance. Recent work by (38) demonstrated that restricting arginine synthesis is a potent inducer of multidrug tolerance in *S. aureus*. The mechanism identified was the induction of the *relA*-dependent stringent response, leading to the inhibition of protein synthesis. Complementing the synthesis branch, the arginine deiminase (ADI) pathway encoded by the *arc* operon (including *arcB* and *arcC*) functions as an active metabolic stress-response module with multifaceted roles. In stressful and acidic biofilm environments, ADI pathway activation is essential for *Streptococcus pyogenes* tolerance, as it produces ammonia to buffer intracellular pH (61). This protective function is conserved, as activating the ADI pathway in *Enterococcus faecalis* and *Streptococcus mutans* also increases antibiotic tolerance (62, 63). These studies often link *arc* upregulation to active defense, whereas our proteomic data identified *arcA* downregulation in our tolerant strains (Table 1). We propose that in the nutrient-rich TSB medium used in our study, the evolved strains achieve the pro-tolerant physiological state via a different metabolic tactic: downregulating the catabolic *arcA* pathway to conserve the internal arginine pool. Given that arginine is also a precursor for host nitric oxide synthesis, this metabolic node likely represents a critical and complex interface between bacterial metabolism, redox sensing, and the host immune response *in vivo* (64)

### Broader context: endogenous ROS in tolerance, host-pathogen interactions, and the antibiotic-induced ROS debate

The role of ROS in antibiotic action has been a subject of significant debate. Seminal work by Kohanski et al. proposed that ROS production is a common downstream mechanism mediating the lethality of diverse bactericidal antibiotics, suggesting that antibiotics stimulate metabolic activity leading to ROS generation that damages cellular components (22). While this model gained considerable traction, it has been challenged by studies such as Keren et al., who found no universal correlation between ROS levels and antibiotic lethality, arguing that primary drug-target interactions remain the principal drivers of cell death (65).

While the debate concerning the role of ROS in direct antibiotic-mediated killing continues, our work, alongside compelling studies focusing on host-pathogen interactions, highlights a distinct but critical role for ROS in modulating antibiotic tolerance. For instance, Rowe et al. demonstrated that host-derived ROS generated during respiratory burst can induce antibiotic tolerance in *S. aureus* during systemic infection (48). Similarly, Peyrusson et al. showed that host cell oxidative stress levels directly influence the dormancy and resuscitability of intracellular *S. aureus* persisters (49). These studies powerfully illustrate how the host oxidative environment shapes bacterial tolerance. However, this environment is a battlefield where the function of ROS is highly context-dependent. In the phagocytes, high-concentration host-derived ROS (H_2_O_2_ and HOCl) function as a direct bactericidal weapon. In this scenario, *S. aureus* must employ active defense. In a previous study, the SaeRS two-component system regulates factors that actively reduce the ROS generated by neutrophils, serving as a critical immune evasion strategy (66). This highlights a sophisticated, dual functionality of redox regulation.

Our findings complement these *in vivo* studies by revealing an endogenous pathway to tolerance, distinct from the exogenous, high-stress responses seen during host-pathogen interactions. Our current study provides this complementary perspective, focusing on the role of endogenous ROS homeostasis, which we demonstrate is modulated by the expression of core metabolic genes. This distinction underscores the critical need to interpret ROS-mediated mechanisms within their specific environmental context. It is highly plausible that both pathways, the response to exogenous host ROS and the strategically managed endogenous ROS identified in our work, converge on similar downstream cellular states that promote tolerance. The link we established between core metabolic gene expression, intrinsic ROS levels, and tolerance thus suggests an inherent bacterial pathway capable of inducing this pro-tolerance state.

### Limitations of the study

While this study provides significant insights into the metabolic and redox adaptations underlying antibiotic tolerance in *S. aureus*, certain limitations should be acknowledged. First, although we demonstrate a pro-tolerance effect of the elevated ROS levels observed in our evolved strains, the precise downstream molecular pathways activated by these ROS signals that lead to tolerance remain to be elucidated. Further research is also needed to define the differential ROS thresholds that distinguish between pro-tolerance signaling and overt oxidative damage leading to cell death. Second, the upstream genetic or regulatory factors initiating the differential expression of the identified core metabolic proteins in the evolved tolerant strains were not investigated in the present study. Uncovering these initial triggers and regulatory networks is an important avenue for future research. Third, our experiments were conducted in the nutrient-rich TSB medium. As highlighted by our discussion of arginine metabolism, the metabolic state and adaptive strategies of bacteria are highly dependent on the nutritional context. Therefore, the specific proteomic profiles observed here may differ under the nutrient-limited conditions often encountered *in vivo* or within biofilms. Finally, the assessment of the cellular redox state relied on the DCFH-DA probe. This probe has known limitations regarding its specificity and can be influenced by factors other than the typical ROS, including certain metal ions and the overall peroxidase activity of the cell (67). The use of a scavenger cocktail containing both thiourea (a general ROS scavenger) and bipyridine (a metal chelator) in some experiments limits the ability to definitively attribute the observed phenotypic effects solely to ROS scavenging versus metal ion chelation. Therefore, while our collective data strongly indicate a functional link between the identified core genes, the tolerance phenotype, and an altered cellular redox state, the precise nature of this redox imbalance warrants confirmation with more methodologies in future studies.

## MATERIALS AND METHODS

### Bacteria growth conditions

The bacterial strains and plasmids used in this study are listed in Table S1. *S. aureus* Newman served as the parent strain for antibiotic tolerance evolution experiments. All *S. aureus* and *E. coli* strains were stored in 25% glycerol broth at −80°C and subcultured before use in any experiments. *S. aureus* strains were grown in Trypticase soy broth (TSB) medium at 200 rpm or on TSA plates (TSB with 15 g/L agar) at 37°C. *E. coli* strains were grown in LB medium (10 g tryptone, 5 g yeast extract, and 5 g NaCl per liter) at 200 rpm or on LBA plates (LB with 15 g/L agar) at 37°C. When necessary, different antibiotics were added to the media: 100 μg/mL ampicillin or carbenicillin sulfate for *E. coli* and 10 μg/mL chloramphenicol or 1 μg/mL anhydrotetracycline for *S. aureus*.

### Vectors and strains construction

The *S. aureus* knockout vector pKZ2 was used to generate the corresponding knockout strains, while the expression vector pSE1 was used to create gene overexpression strains. Plasmids were constructed using specific primer pairs. Briefly, target gene fragments from the *S. aureus* Newman genome were amplified using the corresponding primer sets (Table S2). PCR products were ligated into plasmid pSE1 (BamHI/EcoRI) or pKZ2 (KpnI/EcoRI) via Gibson assembly to generate recombinant plasmids. These plasmids were introduced into *E. coli* DH5α (grown in LB medium with 100 μg/mL ampicillin or carbenicillin) through calcium chloride transformation. All *E. coli-S. aureus* shuttle plasmids were first purified in *E. coli* strain IM08B before being electroporated into the target bacterial strains. For gene overexpression, coding sequences were cloned into the pSE1 vector, placing them under the control of the strong, constitutive *S. aureus mecA* promoter (PmecA) derived from pCL15. This promoter drives gene expression under the standard growth conditions used and does not require specific chemical induction.

The double homologous recombination method was used to generate gene knockout mutants, as previously described (68). Stationary phase cultures of *S. aureus* containing the corresponding pKZ2 plasmid were incubated at 43°C to induce homologous recombination (TSB medium supplemented with 10 μg/mL chloramphenicol). Succussed strains were then incubated at 30°C to facilitate plasmid loss (TSA plates supplemented with 1 μg/mL anhydrotetracycline). Single clones were confirmed by PCR and sequencing to check successful gene knockout and subsequently used for further experiments.

### *In vitro* adaptive evolution experiment

The cyclic antibiotic exposure method for tolerance induction was previously described (69). Briefly, overnight bacterial cultures were resuspended in 50 mL of TSB supplemented with a high concentration of antibiotics (20× MIC) and incubated at 37°C with shaking for 3, 5, or 8 h. The exposed bacteria were then pelleted, washed, and resuspended using fresh TSB. After overnight growth at 37°C with shaking, 500 μL cultures were taken for the next cycle of antibiotic exposure. Resistance and tolerance phenotypes were assessed through minimum inhibitory concentration (MIC) determination and antibiotic survival assays.

### Nomenclature of evolution strains

Antibiotic abbreviations were used to indicate independently evolved populations of different antibiotics. The first part of the tolerant strain name denotes the antibiotic type of treatment, the middle part means cycles for antibiotic exposure, while the last part indicates the duration time of the antibiotic (time = 3, 5, and 8 h) in the evolutionary protocol (e.g., CIP-P17-8 indicates an isolate extracted from the 17th passage populations with 8 h ciprofloxacin incubation in each cycle).

### MIC determination for parental strains

The MIC of each evolved strain was determined using the broth microdilution method. Three morphologically similar colonies from each strain were resuspended in TSB and incubated overnight. The overnight cultures were then inoculated at a 1:100 ratio in fresh TSB and further diluted 40-fold in Mueller-Hinton 2 broth (Sigma-Aldrich Co.) containing antibiotics at concentrations ranging from 64 mg/L to 0.03125 mg/L. After 24 h of incubation at 37°C, the lowest antibiotic concentration that completely inhibited bacterial growth was recorded as the MIC value. The MIC of *S. aureus* Newman is listed in Table S3.

### qRT-PCR

Ten milliliters of specially treated overnight cultures was collected for the determination of steady-state mRNA levels. RNA isolation was performed using the Total RNA Kit (TransGen) with an additional lysis step using Lysostaphin (RHAWN Shanghai). RNA was converted to cDNA using the One-Step DNA Removal and cDNA Synthesis SuperMix (TransGen). Quantitative real-time RT-PCR analysis was performed using a StepOne Real-time PCR System (CFX384, BIO-RAD) with Tip Green qPCR SuperMix (TransGen). Three biological samples were assayed in triplicate with the primers listed in Table S2. Relative transcript levels were determined by the comparative threshold (ΔΔCt) method. Data and statistical analysis were performed using GraphPad Prism software.

### Antibiotic survival assays

For the antibiotic survival assay, a colony of each strain was inoculated in TSB medium and cultured overnight. When necessary, ROS scavengers (100 mM thiourea and 1 mM bipyridyl) were added with colony to the TSB at the same time. The overnight cultures (20 µL) were then diluted into 2 mL of fresh TSB supplemented with the respective antibiotics at 20× MIC. At each time point, aliquots were taken as shown in Fig. S3, serially diluted 10-fold in PBS in 96-well plates, and plated to determine bacterial viability by CFU counts. Survival was calculated as a percentage of the starting inoculum and expressed on a logarithmic scale.

### Calculation of tolerance index

The tolerance index (TI) was calculated by measuring the area between the normalized survival curves of the parental strain and the tolerant strain (the green-shaded region in Fig. S3). The TI was determined using the following equation:


$$TI=\left[log10\left(\frac{{CFU}_{t1}^{tol}/{CFU}_{t0}^{tol}}{{CFU}_{t1}^{wt}/{CFU}_{t0}^{wt}}\right)+log10\left(\frac{{CFU}_{t2}^{tol}/{CFU}_{t0}^{tol}}{{CFU}_{t2}^{wt}/{CFU}_{t0}^{wt}}\right)+log10\left(\frac{{CFU}_{t3}^{tol}/{CFU}_{t0}^{tol}}{{CFU}_{t3}^{wt}/{CFU}_{t0}^{wt}}\right)\right]\times \frac{\Delta t}{2}$$

where tol and wt represent the tolerant strain and the parental strain, respectively, and ∆*t* is the difference between the two moments.

To compare the tolerance of different strains against various antibiotics, the relative TI rate was calculated. This rate is the TI of the corresponding mutant divided by the TI of its homologous tolerant strain, with the TI of responding tolerant strains set to one. This method helps control for variations due to genetic background or antibiotic treatments.

### Proteomics analysis

To achieve complete disruption of the collected bacterial cells, the samples were processed using bead beating under low-temperature conditions. Overnight *S. aureus* cultures were diluted in 900 μL of lysis buffer containing 700 μL RIPA lysis buffer (Solarbio, China), 8 M urea, 0.157 mg PMSF (Solarbio, China), and 2 mg lysozyme (Solarbio, China). An equal ratio of bacterial pellet (1:1 vol/wt) and glass beads (Solarbio, China) was added to each tube. Bead beating was performed using a ThermoMixer C (Eppendorf, Germany) at 2,000 rpm for 90 min. Following this, the samples were centrifuged at 20,000 × *g* for 5 min at 4°C to remove cell debris and foam. Protein concentrations were then determined using a BCA kit (Solarbio, China), and the supernatant was transferred to a new tube for further analysis.

For trypsin digestion, the protein solution was reduced with 25 mM dithiothreitol (Sigma) for 1 h at 37°C and alkylated with 50 mM iodoacetamide (Sigma) for 30 min at 37°C in darkness. Proteins were precipitated overnight at −20°C using 5 volumes of cold acetone and then centrifuged. The protein pellet was resuspended in 100 µL of 50 mM triethylammonium bicarbonate. Trypsin was added at a 1:50 mass ratio of trypsin to protein and incubated for 16 h at 37°C. The digestion reaction was terminated by adding 1% formic acid.

The DIA-acquired data were quantitatively analyzed using Spectronaut against the UniProt reference proteome for *S. aureus* strain Newman (Proteome ID: UP000006386). Differential expression analysis was conducted with the R package limma. Proteins exhibiting a >1.5-fold change in expression between the tolerant strains and the Newman strain, with an FDR-adjusted *P*-value < 0.05, were considered DEPs (Table S1). Gene ontology enrichment analysis was performed using the R package clusterProfiler. Only GO terms with *P* < 0.05 were considered significantly enriched (Table S2).

### Measurement of intracellular oxidative state

For the detection of the general intracellular oxidative state, the probe DCFH-DA (2,7-dichlorodi-hydrofluorescein diacetate; Thermo Fisher Scientific) was used. We acknowledge that DCFH-DA is a non-specific indicator whose fluorescence can be influenced by various reactive species and requires factors such as peroxidases or redox-active metals for oxidation, particularly by H_2_O_2_ (70). Therefore, changes in DCFH-DA fluorescence are interpreted cautiously as reflecting alterations in the general cellular redox environment, which may involve, but are not limited to, specific ROS. Additionally, to avoid potential confounding effects from antibiotic selection pressure during the assay preparation, chloramphenicol was not included in the overnight TSB culture immediately preceding cell harvesting for any strains used for the DCFH-DA measurements. Overnight culture was collected and normalized to OD_600_ = 0.5 using PBS containing 10 μM DCFH-DA. After incubating at 37°C for 30 min in the dark, samples were centrifuged and collected (1,400 × *g*, 5 min), then washed with PBS to remove the residual dye and resuspended in PBS. ROS was detected using the Ex/Em = 488/525 nm by fluorescence microplate reader (Varioskan Flash, Thermo Fisher Scientific). A sample containing *S. aureus* Newman wild-type cells lacking DCFH-DA was included as a control for auto-fluorescence. All tubes with cultures were wrapped with aluminum foil to avoid light.

### Statistical analysis

Statistical analyses were performed and visualized using GraphPad Prism 10. Statistical significance was determined using the one-way ANOVA and considered to be represented by *P*-value of <0.05. All error bars represent the standard deviation (SD) of the mean. Each experiment included at least three biological replicates.

## Data Availability

Due to a critical and irretrievable failure of our laboratory's data storage server, the raw mass spectrometry files (.raw) from this study were permanently lost. While the raw files are no longer available, we provided all the processed data and analysis files, which were included in the supplemental materials, including protein identification results, quantification tables, volcano plots, and any other statistical tests performed on the quantitative data.

## SUPPLEMENTAL MATERIAL

Supplemental material 1 spectrum.01868-25-s0001.docx Fig. S1 to S4; Tables S1 to S3.

Supplemental material 2 spectrum.01868-25-s0002.docx Supplemental table legends.

Data S1 spectrum.01868-25-s0003.xlsx Different expression analysis.

Data S2 spectrum.01868-25-s0004.xlsx Gene Ontology rich by strains.

Data S3 spectrum.01868-25-s0005.xlsx Gene Ontology rich by frequency.

ASM does not own the copyrights to Supplemental Material that may be linked to, or accessed through, an article. The authors have granted ASM a non-exclusive, world-wide license to publish the Supplemental Material files. Please contact the corresponding author directly for reuse.
