# Erratum for Liu et al., “Expression patterns of core metabolic genes and elevated intracellular ROS confer drug tolerance in *Staphylococcus aureus*”

**DOI:** 10.1128/spectrum.00852-26

**Published:** 2026-08-25

**Authors:** Jiahao Liu, Yeming Li, Haodong Liu, Shunhua Yan, Haoyi Jia, Yuxuan Yang, Wenting Hu, Jingjia Wang, Wenfeng Li, Huping Xue, Xin Zhao, Long Li, Pilong Liu

## ERRATUM

Volume 14, no. 3, e01868-25, 2026, https://doi.org/10.1128/spectrum.01868-25. Corresponding author Long Li’s email address should read lilong1101@126.com.

