# Supplementary material for: Expression patterns of core metabolic genes and elevated intracellular ROS confer drug tolerance in Staphylococcus aureus
Source: Microbiol Spectr. 2026 Feb 10;14(3):e01868-25. doi: 10.1128/spectrum.01868-25 (PMC12955431; doi:10.1128/spectrum.01868-25)
Supplement: Supplemental material 1 — Fig. S1 to S4; Tables S1 to S3. [file spectrum.01868-25-s0001.docx]

**Supplementary Information**

*Liu et al.* Expression patterns of core metabolic genes and elevated intracellular ROS confer drug tolerance in *Staphylococcus aureus*

*
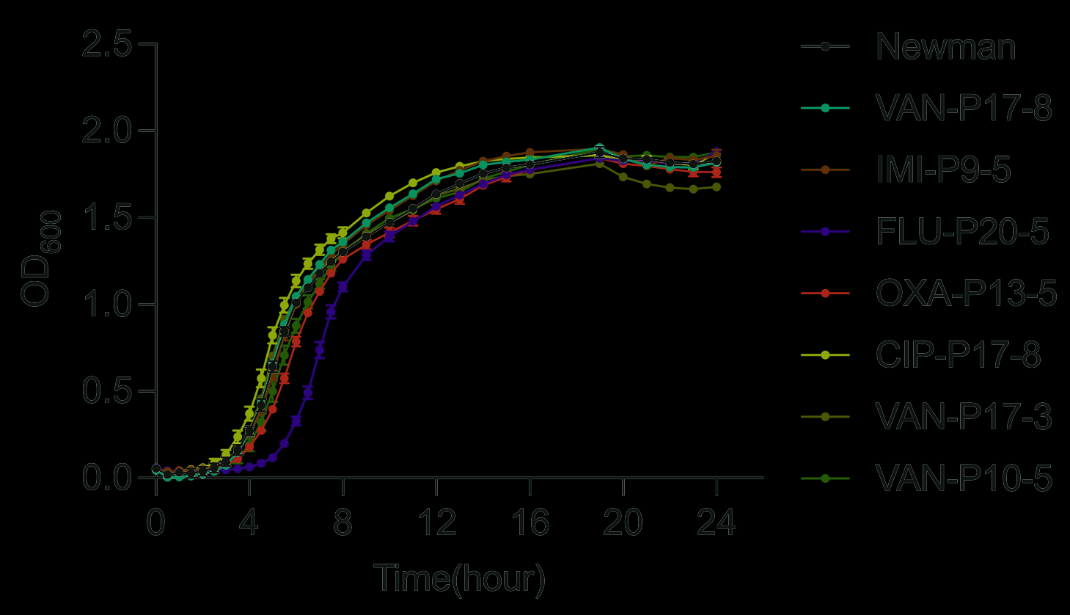
*

**FIG S1 Growth curves of mutants used in this study.** The growth was measured in microtiter plates incubated at 37 °C for 24 h. The curves reflect mean and standard deviations of three biological replicates. Data reflect mean ± SD of three biological replicates.

**
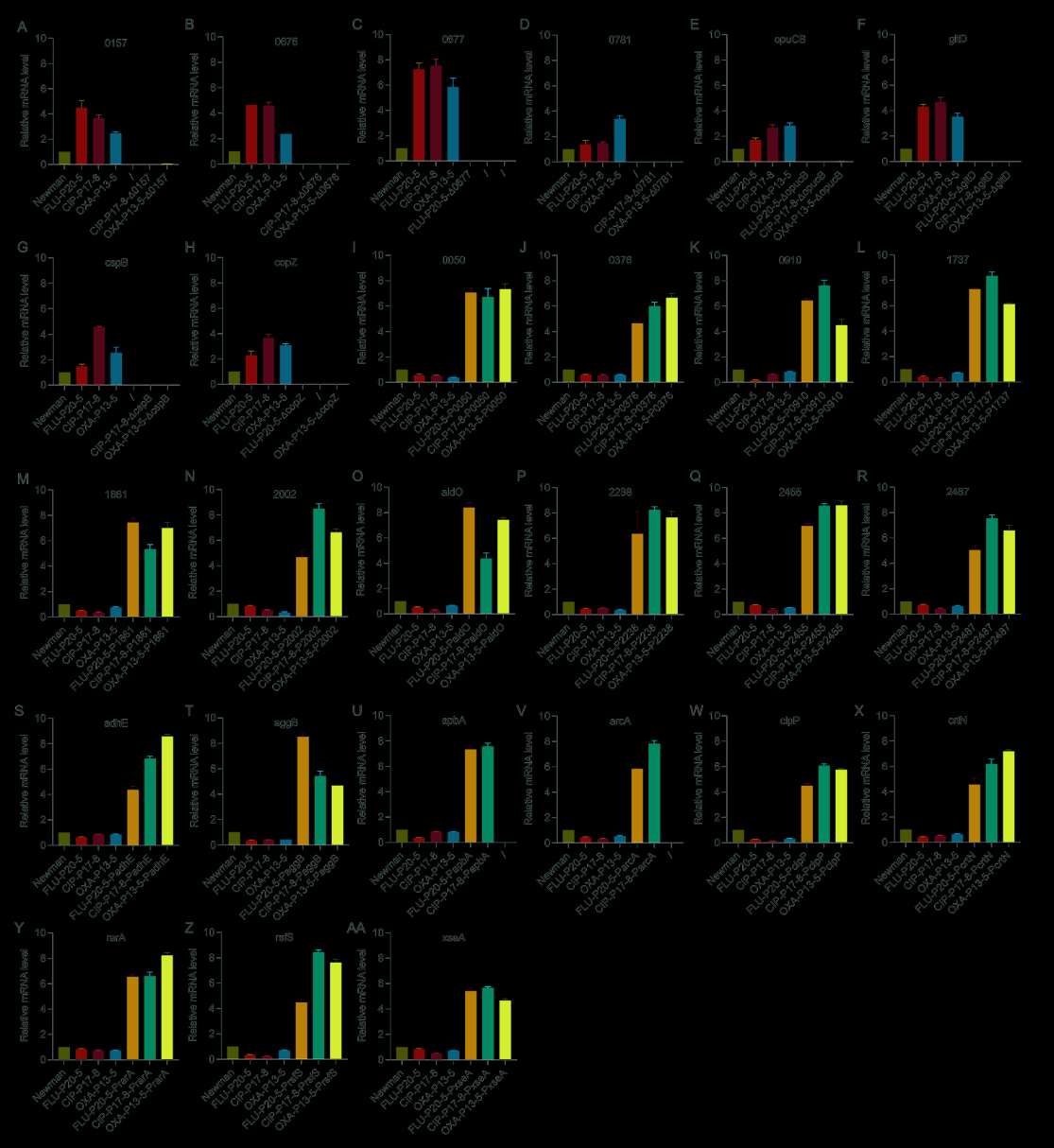
**

**FIG S2 Gene expression level in this study.** The relative mRNA level of 27 genes in tolerant strains and their corresponding knockout or overexpression derivative strains are listed from Fig. S2A to Fig. S2AA. Transcript levels were normalized to 16S genes and presented relative to those of the wildtype strain.


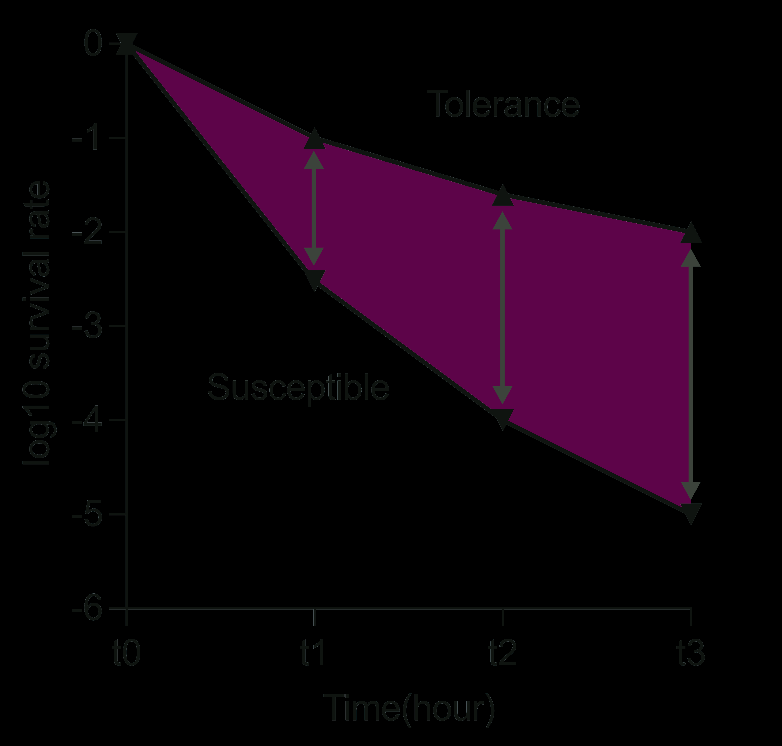


**FIG S3** **Schematic diagram describing the calculation method of Tolerance index (TI).** The area marked in green in Fig. S3 that caught in the middle of susceptive curve and tolerant curve is calculated and used to quantify the TI.

**
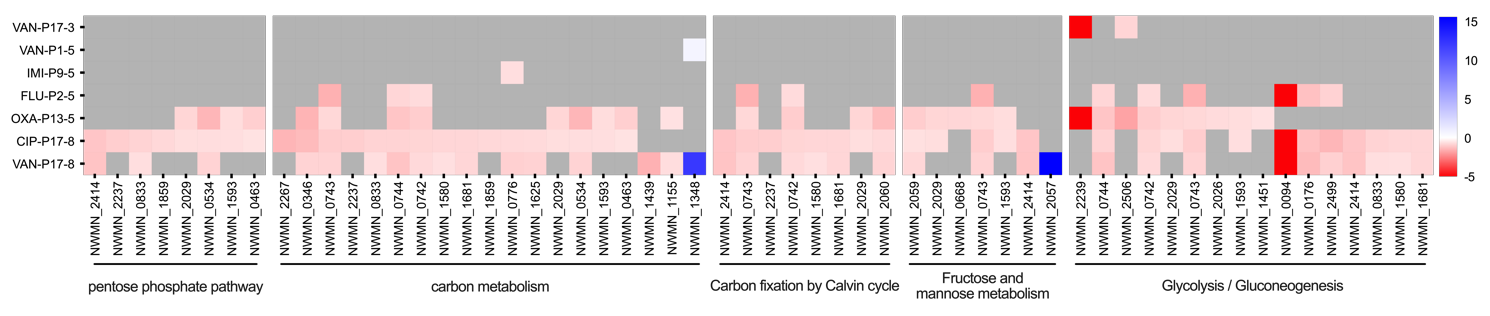
**

**Figure S4. Volcano plot of protein changes related to energy metabolism across seven proteomic profiles.** This volcano plot illustrates the changes in protein expression levels associated with energy metabolism in the proteomic analysis of all tolerant strains. Each column represents an individual sample, while each row corresponds to a specific gene or metabolic pathway. The color gradient, ranging from red to blue, indicates the expression intensity, with red representing low expression and blue representing high expression. The color bar at both ends signifies low (red) and high (blue) expression levels, respectively.

**Supplementary Information Table S1 Isolates and plasmids used in this study**

| Strain and Plasmid | Characteristics | Sources |
| --- | --- | --- |
| Strains |  |  |
| *E. coli* |  |  |
| DH5α | F- endAl glnV44 thi-1 recAl relAl gyrA96 deoR nupG purB20 φ80dlacZ∆M15 ∆ (lacZYA-argF) U169, hsdRl7 (r_K_^-^ m_K_^+^）, λ ^-^ | Lab stocks |
| IM08B | DC10B with staphylococcal (CC8-2)-type methylation system integrated between atpI and gidB genes and (CC8-1)-type between essQ and cspB genes | (1) |
| *S. aureus* |  |  |
| Newman | wild type, human clinical isolate | Lab stocks |
| OXA-P13-5 | Newman with a single mutation within *gdpP* G282D | (2) |
| FLU-P20-5 | Newman with a single mutation within *NWMN_0545* M31I | (2) |
| IMI-P9-5 | Newman with a single mutation within *pth* K127E | (2) |
| VAN-P17-3 | Newman with a single mutation within *stpI* M99V | (2) |
| VAN-P17-8 | unknown | (2) |
| VAN-P10-5 | Newman with two single mutations within *pbp4* T101A and *pth* K127E | (2) |
| CIP-P17-8 | Newman with a single mutation within *savR* D45G | (2) |
| FLU-P20-5-∆0677 | FLU-P20-5 strain with NWMN_0677 deletion | this study |
| FLU-P20-5-∆opucB | FLU-P20-5 strain with opucB deletion | this study |
| FLU-P20-5-∆gltD | FLU-P20-5 strain with gltD deletion | this study |
| FLU-P20-5-∆copZ | FLU-P20-5 strain with copZ deletion | this study |
| FLU-P20-5-P0050 | FLU-P20-5 strain with plasmid pSE0050 | this study |
| FLU-P20-5-P0376 | FLU-P20-5 strain with plasmid pSE0376 | this study |
| FLU-P20-5-P0910 | FLU-P20-5 strain with plasmid pSE0910 | this study |
| FLU-P20-5-P1737 | FLU-P20-5 strain with plasmid pSE1737 | this study |
| FLU-P20-5-P1861 | FLU-P20-5 strain with plasmid pSE1861 | this study |
| FLU-P20-5-P2002 | FLU-P20-5 strain with plasmid pSE2002 | this study |
| FLU-P20-5-PaldO | FLU-P20-5 strain with plasmid pSEaldO | this study |
| FLU-P20-5-P2238 | FLU-P20-5 strain with plasmid pSE2238 | this study |
| FLU-P20-5-P2455 | FLU-P20-5 strain with plasmid pSE2455 | this study |
| FLU-P20-5-P2487 | FLU-P20-5 strain with plasmid pSE2487 | this study |
| FLU-P20-5-PadhE | FLU-P20-5 strain with plasmid pSEadhE | this study |
| FLU-P20-5-PaggB | FLU-P20-5 strain with plasmid pSEaggB | this study |
| FLU-P20-5-PapbA | FLU-P20-5 strain with plasmid pSEapbA | this study |
| FLU-P20-5-ParcA | FLU-P20-5 strain with plasmid pSEarcA | this study |
| FLU-P20-5-PclpP | FLU-P20-5 strain with plasmid pSEclpP | this study |
| FLU-P20-5-PcrtN | FLU-P20-5 strain with plasmid pSEcrtN | this study |
| FLU-P20-5-PrarA | FLU-P20-5 strain with plasmid pSErarA | this study |
| FLU-P20-5-PrsfS | FLU-P20-5 strain with plasmid pSErsfS | this study |
| FLU-P20-5-PxseA | FLU-P20-5 strain with plasmid pSExseA | this study |
| CIP-P17-8-∆0157 | CIP-P17-8 strain with 0157 deletion | this study |
| CIP-P17-8-∆0676 | CIP-P17-8 strain with 0676 deletion | this study |
| CIP-P17-8-∆0781 | CIP-P17-8 strain with 0781 deletion | this study |
| CIP-P17-8-∆opucB | CIP-P17-8 strain with opucB deletion | this study |
| CIP-P17-8-∆cspB | CIP-P17-8 strain with cspB deletion | this study |
| CIP-P17-8-∆gltD | CIP-P17-8 strain with gltD deletion | this study |
| CIP-P17-8-P0050 | CIP-P17-8 strain with plasmid pSE0050 | this study |
| CIP-P17-8-P0376 | CIP-P17-8 strain with plasmid pSE0376 | this study |
| CIP-P17-8-P0910 | CIP-P17-8 strain with plasmid pSE0910 | this study |
| CIP-P17-8-P1737 | CIP-P17-8 strain with plasmid pSE1737 | this study |
| CIP-P17-8-P1861 | CIP-P17-8 strain with plasmid pSE1861 | this study |
| CIP-P17-8-P2002 | CIP-P17-8 strain with plasmid pSE2002 | this study |
| CIP-P17-8-PaldO | CIP-P17-8 strain with plasmid pSEaldO | this study |
| CIP-P17-8-P2238 | CIP-P17-8 strain with plasmid pSE2238 | this study |
| CIP-P17-8-P2455 | CIP-P17-8 strain with plasmid pSE2455 | this study |
| CIP-P17-8-P2487 | CIP-P17-8 strain with plasmid pSE2487 | this study |
| CIP-P17-8-PadhE | CIP-P17-8 strain with plasmid pSEadhE | this study |
| CIP-P17-8-PaggB | CIP-P17-8 strain with plasmid pSEaggB | this study |
| CIP-P17-8-PapbA | CIP-P17-8 strain with plasmid pSEapbA | this study |
| CIP-P17-8-ParcA | CIP-P17-8 strain with plasmid pSEarcA | this study |
| CIP-P17-8-PclpP | CIP-P17-8 strain with plasmid pSEclpP | this study |
| CIP-P17-8-PcrtN | CIP-P17-8 strain with plasmid pSEcrtN | this study |
| CIP-P17-8-PrarA | CIP-P17-8 strain with plasmid pSErarA | this study |
| CIP-P17-8-PrsfS | CIP-P17-8 strain with plasmid pSErsfS | this study |
| CIP-P17-8-PxseA | CIP-P17-8 strain with plasmid pSExseA | this study |
| OXA-P13-5-∆0157 | OXA-P13-5 strain with 0157 deletion | this study |
| OXA-P13-5-∆0676 | OXA-P13-5 strain with 0676 deletion | this study |
| OXA-P13-5-∆0781 | OXA-P13-5 strain with 0781 deletion | this study |
| OXA-P13-5-∆opucB | OXA-P13-5 strain with opucB deletion | this study |
| OXA-P13-5-∆cspB | OXA-P13-5 strain with cspB deletion | this study |
| OXA-P13-5-∆gltD | OXA-P13-5 strain with gltD deletion | this study |
| OXA-P13-5-∆copZ | OXA-P13-5 strain with copZ deletion | this study |
| OXA-P13-5-P0050 | OXA-P13-5 strain with plasmid pSE0050 | this study |
| OXA-P13-5-P0376 | OXA-P13-5 strain with plasmid pSE0376 | this study |
| OXA-P13-5-P0910 | OXA-P13-5 strain with plasmid pSE0910 | this study |
| OXA-P13-5-P1737 | OXA-P13-5 strain with plasmid pSE1737 | this study |
| OXA-P13-5-P1861 | OXA-P13-5 strain with plasmid pSE1861 | this study |
| OXA-P13-5-P2002 | OXA-P13-5 strain with plasmid pSE2002 | this study |
| OXA-P13-5-PaldO | OXA-P13-5 strain with plasmid pSEaldO | this study |
| OXA-P13-5-P2238 | OXA-P13-5 strain with plasmid pSE2238 | this study |
| OXA-P13-5-P2455 | OXA-P13-5 strain with plasmid pSE2455 | this study |
| OXA-P13-5-P2487 | OXA-P13-5 strain with plasmid pSE2487 | this study |
| OXA-P13-5-PadhE | OXA-P13-5 strain with plasmid pSEadhE | this study |
| OXA-P13-5-PaggB | OXA-P13-5 strain with plasmid pSEaggB | this study |
| OXA-P13-5-PclpP | OXA-P13-5 strain with plasmid pSEclpP | this study |
| OXA-P13-5-PcrtN | OXA-P13-5 strain with plasmid pSEcrtN | this study |
| OXA-P13-5-PrarA | OXA-P13-5 strain with plasmid pSErarA | this study |
| OXA-P13-5-PrsfS | OXA-P13-5 strain with plasmid pSErsfS | this study |
| OXA-P13-5-PxseA | OXA-P13-5 strain with plasmid pSExseA | this study |
| Plasimd |  |  |
| pKZ2 | *S. aureus* shuttle vector modified from pKOR1 | (3) |
| pKZ∆0157 | Plasmid used for NWMN-0157 deletion in CIP-P17-8 and OXA-P13-5 strain | this study |
| pKZ∆0676 | Plasmid used for NWMN-0676 deletion in CIP-P17-8 and OXA-P13-5 strain | this study |
| pKZ∆0677 | Plasmid used for NWMN-0677 deletion in FLU-P20-5strain | this study |
| pKZ∆0781 | Plasmid used for NWMN-0781 deletion in CIP-P17-8 and OXA-P13-5 strain | this study |
| pKZ∆opucB | Plasmid used for opucB deletion in FLU-P20-5, CIP-P17-8 and OXA-P13-5 strain | this study |
| pKZ∆cspB | Plasmid used for ccspB deletion in CIP-P17-8 and OXA-P13-5 strain | this study |
| pKZ∆gltD | Plasmid used for gltD deletion in FLU-P20-5, CIP-P17-8 and OXA-P13-5 strain | this study |
| pKZ∆copZ | Plasmid used for copZ deletion in FLU-P20-5 and OXA-P13-5 strain | this study |
| pCL15 | *S. aureus* expression vector, Amp^R^, Cm^R^ | (4) |
| pSC1 | *S. aureus* expression vector modified from pCL15 with no promoter, Amp^R^, Cm^R^ | this study |
| pSE1 | *S. aureus* expression vector modified from pCL15 with the promoter of *mecA*, Amp^R^, Cm^R^ | (5) |
| pSE0050 | pSE1 carrying 0050 coding sequence for overexpression | this study |
| pSE0376 | pSE1 carrying 0376 coding sequence for overexpression | this study |
| pSE0910 | pSE1 carrying 0910 coding sequence for overexpression | this study |
| pSE1737 | pSE1 carrying 1737 coding sequence for overexpression | this study |
| pSE1861 | pSE1 carrying 1861 coding sequence for overexpression | this study |
| pSE2002 | pSE1 carrying 2002 coding sequence for overexpression | this study |
| pSEaldO | pSE1 carrying aldO coding sequence for overexpression | this study |
| pSE2238 | pSE1 carrying 2238 coding sequence for overexpression | this study |
| pSE2455 | pSE1 carrying 2455 coding sequence for overexpression | this study |
| pSE2487 | pSE1 carrying 2487 coding sequence for overexpression | this study |
| pSEadhE | pSE1 carrying adhE coding sequence for overexpression | this study |
| pSEaggB | pSE1 carrying 0526 coding sequence for overexpression | this study |
| pSEapbA | pSE1 carrying apbA coding sequence for overexpression | this study |
| pSEarcA | pSE1 carrying arcA coding sequence for overexpression | this study |
| pSEclpP | pSE1 carrying clpP coding sequence for overexpression | this study |
| pSEcrtN | pSE1 carrying crtN coding sequence for overexpression | this study |
| pSErarA | pSE1 carrying rarA coding sequence for overexpression | this study |
| pSErsfS | pSE1 carrying rsfS coding sequence for overexpression | this study |
| pSExseA | pSE1 carrying xseA coding sequence for overexpression | this study |

**Supplementary Information Table S2 The oligonucleotides used in this study**

| **Name** | **Sequence (5'-3')** |
| --- | --- |
| **gene overexpression** | |
| 0050-pSE1-F | GGTACCGAGCTCGGATCCGGTGGATATGTATTACAGTTATGGAAAT |
| 0050-pSE1-R | ATGGATATCTGCAGAATTCGGTTTTCGTTATAACAATTTGTGTTCT |
| 0376-pSE1-F | GGTACCGAGCTCGGATCCATGGAATTTAAAACCATTCAATCTGTC |
| 0376-pSE1-R | ATGGATATCTGCAGAATTCGTTTAAAATGGTTTACGTAAATCCATTTCT |
| 0910-pSE1-F | GGTACCGAGCTCGGATCCGTGATACGTCAAGCACGTCCAGAGG |
| 0910-pSE1-R | ATGGATATCTGCAGAATTCTTATTTAACAATTAAATGATGATACATGTGCT |
| 1737-pSE1-F | GGTACCGAGCTCGGATCCATGGCAGTAAATTTATATGATTATGCAAA |
| 1737-pSE1-R | ATGGATATCTGCAGAATTCTTAGTCAGCGTAAATTTCGTCTAATG |
| 1861-pSE1-F | GGTACCGAGCTCGGATCCGAGATGTGGAATTTTATTAAATGTGTGT |
| 1861-pSE1-R | ATGGATATCTGCAGAATTCGGATTTATGTCCCAGCCTCTAAAT |
| 2002-pSE1-F | GGTACCGAGCTCGGATCCGTCATGAAATGACCTTAAATCTTCTAAATAAT |
| 2002-pSE1-R | ATGGATATCTGCAGAATTCTTACTCTTCTAATTTTTCATCCTTTAACTTTGG |
| aldO-pSE1-F | GGTACCGAGCTCGGATCCAAAGGAGGTTCATTTTATGAATCATATTG |
| aldO-pSE1-R | ATGGATATCTGCAGAATTCTTAAACATCTAATTCTCTGCTTGTAAGTG |
| 2238-pSE1-F | GGTACCGAGCTCGGATCCATGATATCAATTCACGCAATTTCG |
| 2238-pSE1-R | ATGGATATCTGCAGAATTCGAAAGGTAATTATTTCAATGTTCTGTTATACAT |
| 2455-pSE1-F | GGTACCGAGCTCGGATCCGGAGTTGAAAGGTGTAATGACTG |
| 2455-pSE1-R | ATGGATATCTGCAGAATTCCTACTTTATCGTTTCGTCATTTAATGTT |
| 2487-pSE1-F | GTACCGAGCTCGGATCCATGAATGAACAATGGTTAGAGCATT |
| 2487-pSE1-R | CGAATTGGGCCCTCTAGACGTCTTAACTAACTTGTTGTATCTTGT |
| adhE-pSE1-F | GGTACCGAGCTCGGATCCGAGTGGTTGTATATGTTAACTATACCTG |
| adhE-pSE1-R | CTCTAGATGCATGCTCGAGGATTATTGCTTATAATCATATGATGTTTGAATGATAT |
| aggB-pSE1-F | GGTACCGAGCTCGGATCCGAGGCGATAATATGAATTACATTTTAGGAAC |
| aggB-pSE1-R | ATGGATATCTGCAGAATTCGACTTAGTTTAAAATATTTTGCCACTTTGT |
| apbA-pSE1-F | GCCAGTGTGCTGGAATTCATGAAAATTGCAATTGCTGGAT |
| apbA-pSE1-R | CGAATTGGGCCCTCTAGATTAATCCTGTGCGTGTCGC |
| arcA-pSE1-F | TGGGTACCGAGCTCGGATCCATGACAGATGGTCCAATTAAAGTAAA |
| arcA-pSE1-R | GTGTGATGGATATCTGCAGAATTCTTAAATGTCTTCTCTGAATAATGGTTGAC |
| clpP-pSE1-F | CACACTGGCGGCCGCTCGAGATGAATTTAATTCCTACAGTTATTGAAACAAC |
| clpP-pSE1-R | GGCGAATTGGGCCCTCTAGAGAATTATTTTGTTTCAGGTACCATCAC |
| crtN-pSE1-F | GGTACCGAGCTCGGATCCATGAAGATTGCAGTAATTGGTGCAG |
| crtN-pSE1-R | ATGGATATCTGCAGAATTCTTATACGCCCCGCTCAATAT |
| rarA-pSE1-F | GTACCGAGCTCGGATCCGTGAGTACAGAACCATTAGCATC |
| rarA-pSE1-R | CGAATTGGGCCCTCTAGATTACGGCCTTTGTTTAAGTAAGTT |
| rsfS-pSE1-F | CACACTGGCGGCCGCTCGAGATGAATTCACAAGAATTATTAGCAATTGC |
| rsfS-pSE1-R | GGCGAATTGGGCCCTCTAGATTAATACGCAACCTGACTATATGATTCT |
| xseA-pSE1-F | CACACTGGCGGCCGCTCGAGATGTCAGATTATTTAAGTGTTTCAGCT |
| xseA-pSE1-R | GGCGAATTGGGCCCTCTAGACTTTAGTCATTATTACACCTTACTTTCGT |
| **gene deletion and detection** | |
| ∆0157-pKZ2-upF | AGCCTCGGAACCGGTACCCCGATAAATAATGTTGCGATACCA |
| ∆0157-pKZ2-upR | TGACAGGCTTTCATCTTTAGCGTTGATTTTGACAACATAAAGT |
| ∆0157-pKZ2-downF | TGTTGTCAAAATCAACGCAAAGATGAAAGCCTGTCATCAC |
| ∆0157-pKZ2-downR | GGCGGCCGCTCGGAATTCCACCATATACAATGTTGAAGTTACGT |
| 0157-test-F | GTGCCCAGTTATCAATACCG |
| 0157-test-R | CTTGGCCAACAGCCTACT |
| ∆0676-pKZ2-upF | CCTCGGAACCGGTACCCGTTAAGGTCAAAGTTAGTGTCAT |
| ∆0676-pKZ2-upR | GTGAACATCATGACCCACTTACTGATCGTG |
| ∆0676-pKZ2-downF | GTGGGTCATGATGTTCACATTCTTTCTATTTATTGTGT |
| ∆0676-pKZ2-downR | CCGGCGGCCGCTCGGAATTCGAAGAAATGAGGAGTTAGCATGAAT |
| 0676-test-F | CCTCCTCTTATTTTGACCCCT |
| 0676-test-R | ACGTTTTATCATTGCTTGCG |
| ∆0677-pKZ2-upF | CCTCGGAACCGGTACCAAGTGGGTCATCTATTTTTTCACC |
| ∆0677-pKZ2-upR | AGGAGTTAGCTCAATTTCTGAGTTAAACTTTTATTTACAACATAC |
| ∆0677-pKZ2-downF | CAGAAATTGAGCTAACTCCTCATTTCTTCAATTTG |
| ∆0677-pKZ2-downR | CCGGCGGCCGCTCGGAATTCGGCATCAAATGCAACATTTATCAT |
| 0677-test-F | ACAACTTCATACATTACTTTGGAGC |
| 0677-test-R | GGCATCATGATATCAAGTACCATG |
| ∆0781-pKZ2-upF | CCTCGGAACCGGTACCGGATTAAGAGCAAAAAGACAAAAAG |
| ∆0781-pKZ2-upR | ACCCCCAATTTACCCATGTCTTAACACCTCC |
| ∆0781-pKZ2-downF | ATGGGTAAATTGGGGGTTTCATTTTATGAAAA |
| ∆0781-pKZ2-downR | CCGGCGGCCGCTCGGAATTCAATGAAGTCTTGAATATCTTTAGATTGTAATACT |
| 0781-test-F | GTCCTTGCTGTAGATCACG |
| 0781-test-R | AGCACGCATGCATATAGG |
| ∆opucB-pKZ2-upF | AGCCTCGGAACCGGTACCGTGATTATCCAGTATACGAAATGCG |
| ∆opucB-pKZ2-upR | CATCTTATGTGGCGTTGGGTTGAGCTACCTCCTTTTTCTG |
| ∆opucB-pKZ2-downF | AAAAGGAGGTAGCTCAACCCAACGCCACATAAGATGC |
| ∆opucB-pKZ2-downR | GGCGGCCGCTCGGAATTCCTGGTACATGTGGAGGTACT |
| opucB-test-F | GTTATGGCAAACATGTTGCTG |
| opucB-test-R | TGCTACCACATATCCTACACG |
| ∆cspB-pKZ2-upF | CCTCGGAACCGGTACCGAATAATTAGACGCAGTGGATTAGA |
| ∆cspB-pKZ2-upR | TTATGTTGTAAGAATAGAAAACCTCCGTGTGC |
| ∆cspB-pKZ2-downF | GAGGTTTTCTATTCTTACAACATAAAACGACTCATTATAAATGA |
| ∆cspB-pKZ2-downR | CCGGCGGCCGCTCGGAATTCTGTCGTATGTCAAAACATTTAGTCAAT |
| cspB-test-F | GCATCGTATGTACCACTTCTTG |
| cspB-test-R | AGCATAGTTGGAAAGCTGG |
| ∆gltD-pKZ2-upF | AGCCTCGGAACCGGTACCGGGGTTATTACAGGTAGTGAGAT |
| ∆gltD-pKZ2-upR | ACCATTTCCATACAAAGACGTATCTCCCCCTTTCCTTAA |
| ∆gltD-pKZ2-downF | AGGAAAGGGGGAGATACGTCTTTGTATGGAAATGGTGGTTACG |
| ∆gltD-pKZ2-downR | GGCGGCCGCTCGGAATTCCTTGTCTAGACAAGTTATGTAAAGGC |
| gltD-test-F | TGTCCTTTCGATGGGCC |
| gltD-test-R | CGCGTTATTACTTAATGCGTC |
| ∆copZ-pKZ2-upF | CCTCGGAACCGGTACCGTAGGCATAGATACTGTTATTGCAG |
| ∆copZ-pKZ2-upR | TATTGCCTAAAGCATTCACCTCCTTTTAGAG |
| ∆copZ-pKZ2-downF | GGTGAATGCTTTAGGCAATATTCAACGTCATCAAC |
| ∆copZ-pKZ2-downR | CCGGCGGCCGCTCGGAATTCAATGTGCCGTCATACTCTC |
| copZ-test-F | TGGTATCATCGCAGTGGC |
| copZ-test-R | CTGGTTATGATGGCTTATCGC |
| **vector identification** | |
| pKZ2-F | AATGTCACTAACCTGCCCCGT |
| pKZ2-R | CCCGATTTAAGCACACCCTTT |
| M13F | GTAAAACGACGGCCAGT |
| pCLF | TATCCTAACAGCACAAGAGC |
| **qRT-PCR** | |
| copZ-qRT-F | AAATAATATTGACGGTGTCACTTCAGC |
| copZ-qRT-R | ATTGCGTCTTTCATTTGAGATACAGC |
| gltD-qRT-F | GTAGACCGTTTGAAGCATCATAAAGC |
| gltD-qRT-R | GGCGTTCCACAATCCATACATCG |
| cspB-qRT-F | ATTCGTACATTTCTCAGGTATCGCTAG |
| cspB-qRT-R | TCCACGTTGACCTTCAGTGATTTC |
| opucB-qRT-F | ATTGAAGCGGCTAAAGGTATAGGTATG |
| opucB-qRT-R | CATTATAACAGGCATTGCTATCGGAAG |
| 0781-qRT-F | ACGATAAGTGGTCCGACAGGTG |
| 0781-qRT-R | CCAAGCCGCTTCGATGACAC |
| 0677-qRT-F | CGCCGAAGATGACGTGAGC |
| 0677-qRT-R | AAGAAATCGCAATGGTTGACTACG |
| 0676-qRT-F | TGGTGAATCAGTATTGTTAGCGATGAC |
| 0676-qRT-R | TTCGTGTGGGTTCAGGTATTGTTATG |
| 0157-qRT-F | ATAAATGCCTCTACACCAGCCAATAC |
| 0157-qRT-R | AGCAGACGGTGCAGTTGAATATG |
| xseA-qRT-F | AATAACAGCAGCTTGAGTTGGAGTC |
| xseA-qRT-R | AAGACACCGATTATATCAGCAGTTGG |
| rsfS-qRT-F | CGCAACCTGACTATATGATTCTAATGG |
| rsfS-qRT-R | AATTGACTTAGCTGATGTTGTGGTAC |
| rarA-qRT-F | ATTCCACTAAGCCAAGCAGTAATCG |
| rarA-qRT-R | GCCCACATGACCGTTTCTAATATCG |
| crtN-qRT-F | AATTGATTCATACGCCCGCCTAC |
| crtN-qRT-R | GCAGGTGTCACAGGATTAGCAG |
| clpP-qRT-F | CGGTATGGCTGCATCAATGGG |
| clpP-qRT-R | CTTGTCCTTGAGCACCACCTAATG |
| arcA-qRT-F | GTCACTACAACGCCTGGTCTTATAC |
| arcA-qRT-R | GGTGATGTCATTGATGGTGCTAGAG |
| apbA-qRT-F | ACCTGCCGTCCAAGTCGTAAC |
| apbA-qRT-R | TGAATGGTCTGAAGCATGAAGAAGTC |
| aggB-qRT-F | CGCTTTCAGTAGGTACACCAGTG |
| aggB-qRT-R | CAACCATTTGATTGATGTCACCTTGAG |
| adhE-qRT-F | AAGCGTTACAGCAGATACAGGAATG |
| adhE-qRT-R | CCTCTTGTGTAGTCTGAAGCCATTAC |
| 2487-qRT-F | TTCTTCTTGTTGCTGACTGTGTAATTG |
| 2487-qRT-R | GTGGCGAGGTTAACGGTGATG |
| 2455-qRT-F | TACGAACGACTTTACAAGGGTTTCC |
| 2455-qRT-R | TGTGATTGGTGCTGGTAGTGTTG |
| 2238-qRT-F | ATAAAGCAGTTTGTGGGTTTAGTAAGC |
| 2238-qRT-R | CATCGCATGAGTAGGTAGTGTAGG |
| aldO-qRT-F | ACGCAGTAGGTGGGCATAATTTATATC |
| aldO-qRT-R | ATGATTAATGGCTTGACGAACAACATC |
| 2002-qRT-F | CACGGGTTGGTTGCTTGAAATTG |
| 2002-qRT-R | GATGACGACACGAATATCCAATAACTG |
| 1737-qRT-F | TGGAAGTTAATTTGAGTTTCACGGAAC |
| 1737-qRT-R | AGAGAAAGCGAAGAATACAAAGCAATC |
| 0910-qRT-F | CCTGTAAAAGAAGCTAAAGACGATGAG |
| 0910-qRT-R | GCGATGCCTCTACCTCTATATGC |
| 0376-qRT-F | TGCGTCATTAACTCATCATGGTTGG |
| 0376-qRT-R | CAACTGCACATTATGAAGCCACAAC |
| 0050-qRT-F | TCTTGGTGGTCATTCAGTTCGTATATG |
| 0050-qRT-R | TCGTGGTATTCCAGAAGTCATCAATAG |
| 1861-qRT-F | TTAGTTATTTTTACTTTCAATATCAGAAATAATTTCTTT |
| 1861-qRT-R | ATGTGGAATTTTATTAAATGTGTGTTTAAATTC |

**Supplementary Information Table S3 The MIC of the antibiotics for *S. aureus* Newman**

| Antibiotic | MIC (mg/L) |
| --- | --- |
| Oxacillin | 0.25 |
| Flucloxacillin | 0.25 |
| Imipenem | 0.06 |
| Vancomycin | 2 |
| Ciprofloxacin | 0.25 |

**REFERENCE**

1. Monk IR, Tree JJ, Howden BP, Stinear TP, Foster TJ. 2015. Complete Bypass of Restriction Systems for Major Staphylococcus aureus Lineages. mBio 6:e00308-15.

2. Yu XH, Hao ZH, Liu PL, Liu MM, Zhao LL, Zhao X. 2022. Increased Expression of Efflux Pump norA Drives the Rapid Evolutionary Trajectory from Tolerance to Resistance against Ciprofloxacin in Staphylococcus aureus. Antimicrob Agents Chemother 66:e0059422.

3. Liu P, Wu Z, Xue H, Zhao X. 2017. Antibiotics trigger initiation of SCCmec transfer by inducing SOS responses. Nucleic Acids Res 45:3944-3952.

4. Luong TT, Lee CY. 2007. Improved single-copy integration vectors for Staphylococcus aureus. J Microbiol Methods 70:186-90.

5. Wu Z, Zhang L, Qiao D, Xue H, Zhao X. 2018. Functional Analyses of Cassette Chromosome Recombinase C2 (CcrC2) and Its Use in Eliminating Methicillin Resistance by Combining CRISPR-Cas9. ACS Synth Biol 7:2590-2599.
