# Supplementary material for: Expression patterns of core metabolic genes and elevated intracellular ROS confer drug tolerance in Staphylococcus aureus
Source: Microbiol Spectr. 2026 Feb 10;14(3):e01868-25. doi: 10.1128/spectrum.01868-25 (PMC12955431; doi:10.1128/spectrum.01868-25)
Supplement: Supplemental material 2 — Supplemental table legends. [file spectrum.01868-25-s0002.docx]

Supplementary table legends

Supplementary Table 1 Differentially expressed proteins identified in seven antibiotic tolerant strains compared to wild type.

Supplementary Table 2 Gene ontology rich of DEPs in seven antibiotic tolerant strains.

Supplementary Table 3 DEPs shared across different strains.
